# Developing Iron and Iodine Enrichment in Tomato Fruits to Meet Human Nutritional Needs

**DOI:** 10.3390/plants13233438

**Published:** 2024-12-07

**Authors:** Nabeel Ahmad Ikram, Muna Ali Abdalla, Karl H. Mühling

**Affiliations:** 1 Department of Agronomy, Muhammad Nawaz Shareef University of Agriculture, Multan 60000, Pakistan; nabeel.ahmad@mnsuam.edu.pk; 2 Institute of Plant Nutrition and Soil Science, Kiel University, Hermann-Rodewald-Str. 2, 24118 Kiel, Germany; khmuehling@plantnutrition.uni-kiel.de

**Keywords:** iodine, iron, tomato fruit, biofortification, human health

## Abstract

Iron (Fe) and iodine (I) are essential microelements required for a healthy life, with Fe playing a vibrant role in oxygen transport, and I is vital for cognitive development and thyroid function. Global Fe and I deficiencies affect a significant portion of the population worldwide, leading to widespread health concerns, especially anemia, impaired cognitive function, and thyroid disorders. This review not only inspects the potential of agronomic biofortification to enrich Fe and I content in tomatoes, but also highlights its bright future for crop nutrition. It discusses the latest developments in agronomic biofortification methods focused on improving the enrichment of Fe and I in tomatoes, emphasizing practical approaches such as seed priming, soil application, and foliar spray. Notably, the review explores the promising impacts of Fe and I biofortification on growth, yield, and improved fruit quality in tomatoes. Moreover, it offers an in-depth investigation of the efficacy of agronomic biofortification in enhancing the nutritional contents of tomatoes by combining the most recent research findings. It highlights the impact of agronomic biofortification in mitigating micronutrient deficiencies worldwide and its capacity to encourage sustainable agriculture and improve community health by enhancing crop nutrition.

## 1. Introduction

Micronutrients are key components of balanced nutrition and play a pivotal role in supporting normal growth and development in both plants and humans. Despite increased access to food with a significant reduction in global hunger in the past few decades, deficiency of micronutrients like zinc (Zn), iron (Fe), and iodine (I) poses a serious challenge to human health worldwide [1]. Globally, micronutrient deficiencies are common; among them, I deficiency affects about 21.9% of the population, while Fe deficiency affects about 30% of the population [2]. Inadequate intake of these nutrients causes a wide array of severe health complications, including chronic diseases. The lack of adequate food to fulfill daily nutritional requirements is commonly called malnutrition, the most critical risk factor for disease burden in low-income countries [3]. Most of the population in these countries depends on staple crops (rice, maize, sorghum, and wheat) for their food, which do not provide the essential macro- and micronutrients, ultimately resulting in their deficiency [4].

Micronutrient deficiency is also termed hidden hunger, which occurs when essential minerals are not sufficiently taken up in the routine diet, thus having disturbing impacts on human health, especially in vulnerable populations. For instance, deficiency of I continues to be a global concern for about 2 billion people, highlighting the ongoing public health concerns in developing and developed countries. It was assessed from urinary I data of school-aged children globally that one-third of these children have an inadequate consumption of I in their daily diet [5]. Chronic deficiency of I can trigger growth impairment, goiter, cretinism, intellectual disability, hearing loss, and reproductive failure [6]. I deficiency in men can lead to hypothyroidism, slower mental function, and decreased fertility, including lower sperm count and quality [7]. Additionally, Fe deficiency affects approximately 1.62 billion people worldwide and considerably contributes to anemia [8,9]. Deficiency of Fe continues to pose a serious hazard to child and maternal health, with recent research emphasizing its devastating global impacts. For example, in the USA, Fe deficiency affects almost 10% of pregnant women [7], while in Nigeria, shockingly, 57% of pregnant women suffer from deficiency of Fe [10]; at present, deficiency is also responsible for anemia in 42% of children under five and 40% of pregnant women worldwide [11]. Moreover, Fe deficiency in men can lead to cognitive issues, fatigue, weakness, heart problems, and a weakened immune system [12]. Recent studies continue to highlight the serious health issues of Fe deficiency in Pakistan. For example, a study conducted by [13] stated that deficiency of Fe is the main contributing factor leading to various pregnancy complications among pregnant women in Pakistan, such as low birth weight, anemia, increased risk of preterm birth, and poor fetal development. Likewise, in India, a deficiency of Fe remains the major cause of nutritional anemia, highlighting the urgent need for an effective biofortification program [14]. Furthermore, proper Fe nutrition could overcome anemic conditions caused by Fe deficiency.

Producing crops rich in Fe and I is a major challenge for plant breeders and food crop scientists, as the growing population needs more nutrient-dense foods to combat widespread deficiencies [15]. However, several high-yielding varieties of food crops have been released in recent years, which have somewhat helped us to address the challenge of food security. However, most of the germplasm varieties lack micronutrients (Fe, Zn, and I), which are essential for human health [1]. This ultimately resulted in micronutrient deficiencies, especially of I, Zn, and Fe, and various vitamins [16]. To address these micronutrient deficiencies, various strategies can be employed, including industrial fortification, dietary diversification, biofortification, and mineral supplements [14]. The deficiency symptoms of I and Fe can be corrected through supplementation, diet diversification, or fortification [17]. Industrial fortification is an important strategy, but it is a short-term and cost-intensive approach. Therefore, the research can focus on long-term approaches that will be sustainable and cost-effective. Thus, a strategy for the production of biofortified food crops is useful for promoting food security. Such crops can be developed using conventional plant breeding techniques, crop biotechnology, and agronomic methods to minimize deficiency of Fe and I with increased bioavailability of these nutrients in edible parts of plants [18]. This proves that edible crops can supply various essential nutrients that are necessary for a healthy lifestyle. Ironically, at present, farming systems are more focused on increasing net returns by increasing production per unit area, but not on improving the nutritional value of edible crops [19].

## 2. The Importance of I and Fe for Human and Animal Nutrition and Health Status

Iodine and iron are essential micronutrients that play vibrant roles in human and animal nutrition and health. Iodine is crucial for the synthesis of thyroid hormones [6,20]. Similarly, Fe is also critical for different cellular processes, including proper thyroid functioning, and immune system defense [21,22]. The prevalence of Fe and I deficiency disorders is listed in Table 1. I deficiency is still a public health problem; about one-third of the population around the globe is suffering from this deficiency [23,24]. Deficiency of I is still widespread in some developing regions like Sub-Saharan Africa, South Asia, and parts of Central Asia; in contrast, I deficiency affects a much smaller proportion of the population in developed nations, often less than 5–10% [9]. Insufficient I intake results in a variety of clinical and social concerns that are termed iodine deficiency disorders (IDDs) [25]. These IDDs can lead to infant mortality, neuro-psychological complications, growth impairment, and increased pregnancy loss, which have a great impact on the quality of human life and ultimately affect the economic efficiency of a community [26]. In adults, I deficiency causes intellectual disability and laziness [27]. Moreover, I deficiency is linked to various health problems like goiter, mental/intellectual retardation, stunting, and increased pregnancy and newborn mortality [28].

On the other hand, Fe deficiency is classified as the world’s foremost important nutritional disorder in developed and developing nations [29,30]. Fe deficiency leads to nutritional anemia in children under five and pregnant women, especially at the reproductive stage [31]. Additionally, Fe deficiency results in stunted mental growth and a weakened immune system in humans [32]. The World Health Organization reports that over 60% of the global population suffers from Fe deficiency [29]. The recommended dietary allowance of Fe is 8, 18, 10, and 27 mg per day for adult males, females, kids, and pregnant women, respectively [33]. Fe exists as storage Fe (bound to ferritin), functional Fe (bound to myoglobin, hemoglobin, and enzymes), and transport Fe (transit to tissues) in the human body [31]. Fe deficiency is a significant public health challenge, particularly among women and children [34], and acts as a primary cause of childbirth complications, leading to fetus abortion in women [35]. Patients with Fe deficiency suffer from anemia because their storage, functional, and transport of Fe are depleted [36]. Anemic mothers give birth to children suffering from improper brain function, leading to behavioral disorders, poor physical activity, and impaired learning ability [37]. Edible food items like wheat flour, pasta, macaroni, cornflakes, and puffed rice are fortified with Fe [16]. Fe has good potential of being absorbed in our bodies when taken along with white meat, mutton, or with vitamin C-enriched foods like peppers, broccoli, tomatoes, citrus, and strawberries [38]. The recommended daily allowance of Fe is 11 mg/day for children and 15 mg/day for adults [33].

## 3. Tomato Fruit Is an Ideal Crop for I and Fe Biofortification

Vegetables serve as a vital source of food and play a very important role in disease prevention and human health maintenance. They also serve as a source of minerals, antioxidants, and vitamins. Tomato (*Solanum lycopersicum*) is a member of the family Solanaceae (having a diploid number of chromosomes = 24), and in terms of global production, it serves as the third important vegetable. Botanically speaking, the tomato is a fruit, although in culinary terms, it can also be called a vegetable. Currently, almost 7500 different genotypes of *S. lycopersicum* are under cultivation globally, consumed fresh or in processed form [39]. They are full of beneficial vitamins, minerals, and antioxidants, including lycopene, which has been linked to minimizing the risk of cancer [25]. Tomatoes are also a key component of the Mediterranean diet, which has been shown to lower the risk of cancer and heart problems [40]. They are ranked as the third-most important vegetable crop globally, following potato and onion [41]. They are highly valued for their flavor and nutritional richness, with 100 g of tomato containing approximately 4.56 mg of iron, 166 mg of calcium, and significant amounts of vitamins A, C, and fiber [42]. Due to the rise of the fast food industry, tomatoes have gained much popularity and are cultivated worldwide commercially in different conditions, like in fields, greenhouses, and net houses, and used for both the fresh market and the processing industry [43]. Various factors like environment (temperature, light, mineral, air composition, and nutrition), agronomic practices (fertilization, irrigation, plant protection, and harvest stage), and genetics (variety or cultivar) have a great impact on the nutritional composition of tomatoes [44]. Daily consumption of tomatoes has been associated with reduced risks of inflammatory processes, cancer, and chronic non-communicable diseases like coronary heart disease and diabetes [45]. Despite its natural deficiency in I, tomatoes have shown potential for effective iodine accumulation in their edible fruits through biofortification efforts. Recent studies have demonstrated that agronomic biofortification can increase I content in tomato fruits up to 10 mg of I per kg of fresh fruit weight, which is more than sufficient for fulfilling the daily dietary allowance of I [25,46]. Thus, tomatoes are considered an ideal crop for agronomic biofortification due to their potential role of I enrichment in the human diet.

Encouraging results in terms of effective Fe enrichment up to 7.52 mg per 100 g in tomato fruits were achieved through the enrichment of Fe in the hydroponic system [47]. The major Fe inhibitors that reduce Fe bioavailability are phenolic and phytate compounds [48]. Tomatoes can release ferric reductase enzymes from their roots, which helps in the reduction of Fe^3+^ to Fe^2+^, which is readily taken up by tomato plants. Ferric chelate reductase gene LeFRO1 in tomatoes causes the reduction of chelated ferric Fe (Fe^3+^) into soluble ferrous Fe (Fe^2+^) at the surface of root hairs [49]. Collectively, the I and Fe biofortification in vegetable crops, especially tomatoes, offers a viable strategy to improve the nutritional composition of tomatoes, thus improving public health worldwide. Previous studies of Fe and I biofortification in tomato fruits are listed in Table 2. Hence, tomatoes are consumed globally in both fresh and processed forms. Improved bioavailability of essential nutrients in edible crops that are consumed fresh or cooked, just like tomatoes, can be used as an exact target for agronomic biofortification.

## 4. Iodine and Iron in Plant–Soil System

**Figure 1:**
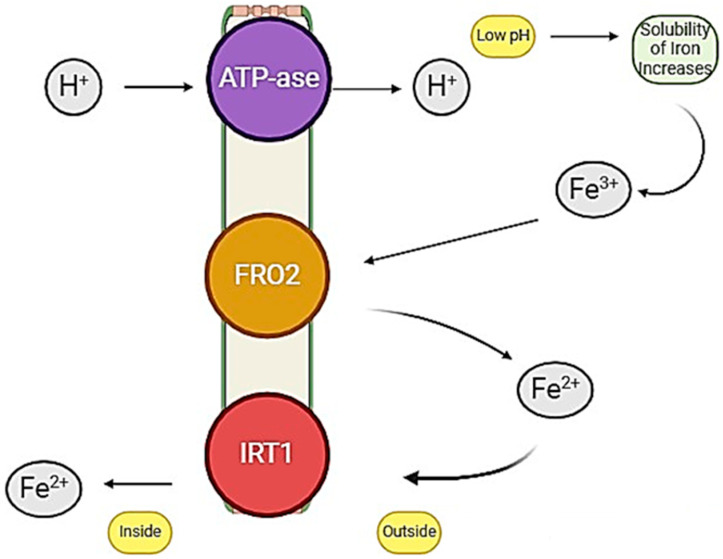
Strategy I for the uptake of “Fe” from the rhizosphere. Iron-regulated transporter 1 (IRT1) facilitates the uptake of ferrous iron (Fe^2+^) into root cells. Ferric Reductase Oxidase 2 (FRO2) converts ferric iron (Fe^3+^) to Fe^2+^ at the root surface, aided by Plasma Membrane H^+^-ATPase (ATPase), which maintains the necessary proton gradient.

Iodine and iron are vital micronutrients beneficial for crop growth and development, and have critical roles in various physiological and biochemical processes taking place in crop plants. Historically, the role of I in plant nutrition has been overlooked, but currently, its involvement in plant biochemical and physiological processes has gained recognition [23]. I is present in the water of seas or oceans, and acts as a source from which I is volatilized into the atmosphere and transported onto soils and plants through aerial deposition or rain. In soil, I exists as inorganic ionic forms like iodate (IO_3_^−^) and iodide (I^−^), as well as in organic forms. Plant behavior is very complex regarding I uptake from the soil because it is dependent on soil–plant interaction, which involves several factors like soil texture, composition, redox conditions, soil pH, etc., having a significant impact on I uptake by plant roots [56]. Field crops have complex behaviors in I uptake, which depend intricately upon soil–crop interactions. It is not easy to distinguish between essential and beneficial nutrients, particularly when it comes to trace elements like I [57]. However, more recent research by [58,59] elaborated I as a beneficial but non-essential nutrient, and its inclusion in the list of beneficial nutrients today reflects the change in previously set criteria and standards [60]. Recent research findings depicted that agronomic biofortification of I at optimal concentration has positive impacts on the growth of tomatoes, strawberries, and cabbage [61]. I application at optimal concentrations can improve the antioxidant response in crops, which protects against different abiotic stresses like salinity or heavy metals stress [62]. Agronomic biofortification of I leads to effective I enrichment in edible parts of crop plants [46]. However, I application at a higher rate impedes its absorption by plant roots [63]. In contrast, not only is Fe is an essential micronutrient, but also, it acts as a signaling molecule playing a key role in critical cellular processes [64]. Moreover, it serves as a cofactor in various enzymatic reactions and plays a crucial role in various biochemical, chemical, and physiological processes like oxygen transport, photosynthesis, and respiration [52]. In an alkaline environment and at a neutral pH, the ferric form of Fe has a low solubility, which ultimately results in the appearance of Fe deficiency symptoms in crops, especially at critical growth stages; to increase Fe solubility, acidification or chelation practice in the root zone is necessary [65]. Plants use various approaches for the uptake of Fe from the soil; however, reducing the risk of increased Fe concentration in cells may lead to oxidative stress. Plants use two different strategies, known as Strategy I and Strategy II, to mobilize Fe from the rhizosphere. In general, non-graminaceous monocots and dicots utilize Strategy I, while all other monocots adopt Strategy II for Fe uptake from the rhizosphere through their root hairs [66]. In plants using Strategy I, rhizosphere acidification is mediated by proton extrusion by the plasma membrane H^+^ ATPase 2 “AHA2” [67]. Under iron-deficient conditions, “AHA2” transcript accumulation to higher levels argues for its contribution to acidification-driven Fe uptake. Briefly, soil acidification facilitates the dissolution of ferric iron (Fe^3+^) precipitates and increases Fe availability [68]. Strategy I regarding the uptake of Fe from the rhizosphere is presented in Figure 1.

Strategy II plants, which include grasses, release specific compounds during Fe deficiency in the soil, known as phytosiderophores. These compounds are nonprotein amino acids that are synthesized from methionine to solubilize and chelate inorganic Fe^3+^ for efficient uptake from soil [65]. Explicitly, a class of coumarin-type siderophores from the phenylpropanoid pathway released in the rhizosphere via an ABC-type transporter aids in membrane-bound acidification and ferric reduction by solubilizing and reducing Fe from insoluble sources [69]. Coumarins not only directly contribute to increased iron availability, but in many cases, also alter the root microbiota-mediated iron solubilization process, allowing plants to utilize the limited Fe supply in the soil [70]. Excess of Fe in the form of Fe^3+^ can be toxic for plants; hence, there is a basic need to convert this form into a soluble form like Fe^2+^. In the root zone, Fe^3+^ is first reduced to Fe^2+^; then, it can be taken up by plants. However, an excess amount of Fe^2+^ can react with oxygen and produce highly reactive free radicals, which ultimately damage the cellular organelles and cells. Thus, to prevent this damage, excessive Fe is chelated by citrate and transformed and further stored in the form of ferritin molecules for future plant needs [71]. Understanding the basic dynamics of crops, I and Fe uptake within the soil environment is essential for enhancing universal food security and augmenting agricultural practices to ensure adequate micronutrient availability for proper plant growth. Incessant studies into the complex interactions among plant physiology, plant micronutrient uptake, and soil properties will further improve agronomic biofortification plans for sustainable and nutritionally enriched tomato production.

## 5. Iodine and Iron Biofortification Strategies

To address widespread hidden hunger, particularly due to I and Fe deficiencies, crop scientists must focus on crop biofortification strategies to promote food and nutrition security. Hidden hunger refers to the deficiency of essential micronutrients, which means a particular nutrient is not adequate in the daily diet. Crop biofortification has two basic strategies: genetic biofortification and agronomic biofortification. Genetic biofortification of crops involves both conventional breeding and transgenic approaches. Conventional breeding is the most acceptable form of genetic biofortification because of its great acceptability and sustainability. This approach identified germplasm with higher nutrient value, which was then crossed with high-yielding but relatively less nutrient-rich genotypes [72]. A huge number of programs and projects, like the Health Grain Project and Harvest Plus, have mainly supported the biofortification of various edible crops like wheat, corn, and millet through conventional breeding. But sometimes, conventional breeding programs may be very challenging due to the lack of exploitable genetic variability in the gene pool, so a long time is needed for the introgression of a desirable trait into commercial cultivars, often accompanied by linkage drag [73]. Therefore, breeding to improve nutrient content is expensive and time-consuming. However, the transgenic approach, which involves nutrient-based gene identification, its transfer, and expression to enrich crops with specific nutrients, has significant advantages. Researchers have identified various genes controlling the availability of certain essential micronutrients in edible parts of crops like “Flavr Savr”, which is a genetically modified tomato having resistance to tomato rotting [74], and pro-vitamin A-enriched “Golden rice” [75]. In addition to consumer acceptability and the complex regulations of commercialization, the transgenic approach is highly cost-intensive [76].

**Figure 2:**
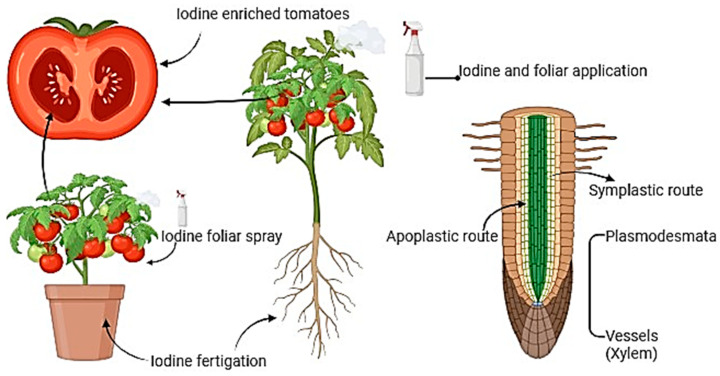
The “I” uptake and its mobilization pattern in tomatoes plants.

Keeping in view the limitations of genetic biofortification, agronomic biofortification may be used as an affordable alternative. It is an encouraging and cost-effective strategy for enhancing nutrient contents in edible crops through seed priming techniques, soil amendments, and foliar sprays [18]. Agronomic biofortification of crops has been employed as a promising tactic to improve I and Fe contents in edible crops, especially leafy vegetables, and tomatoes, to overcome human micronutrient deficiencies. Biofortification strategies like soil application of potassium iodide (I^−^) or potassium iodate (KIO_3_) have demonstrated remarkable results [77,78]. The efficacy of I biofortification depends upon suitable crop cultivars with efficient I enrichment ability, the use of affordable methods for widespread application, and the management of the environmental impacts of excessive I application [78,79]. For example, I biofortification of tomatoes through fertigation or foliar application showed a substantial increment in the fruit’s I content without compromising crop yield [46,79]. The I uptake and its mobilization pattern in tomato plants are depicted in Figure 2. Research findings demonstrated that vegetables like celery, peppers, and radishes are hyperaccumulators of I, when I is applied via nutrient solution or soil application, therefore economically addressing I deficiency [80,81].

Likewise, the efficacy of Fe biofortification, which aims to combat Fe deficiency globally, is hindered by several factors. The majority of the Fe in plant-based foods is non-heme Fe, which is less available than heme Fe present in animal-based foods [82]. Careful optimization of agronomic techniques such as nutri-seed priming, soil application/fertigation, and foliar application, along with their application rates, is necessary since high Fe concentrations can occasionally have a detrimental effect on plant growth and yield [83]. It still needs to be carried out to consistently raise the Fe content of many crop cultivars [84]. Environmental factors are also crucial since the overuse of Fe in agriculture can cause pollution and ecological disruptions [48]. To overcome these challenges, more studies are needed to increase the understanding of Fe absorption and transport mechanisms in plants, investigate new iron-enriching methods, and optimize agronomic approaches [85]. Agronomic approaches for iron biofortification involve soil applications of iron sulfate (FeSO4) or iron chelates to enhance iron uptake by crops like wheat, chickpeas, and rice [48,84]. While FeSO_4_ is widely used, its effectiveness can be influenced by soil pH. Low pH improves its solubility, leading to promoted Fe uptake by plants, but at high pH (in alkaline soils), it precipitates and becomes less available [86]. On the other hand, in comparison to FeSO_4_, Fe chelates such as EDTA-Fe are more expensive, but they are more stable in a wider range of pH and ideal for soils with high pH, making them a better option in alkaline soils [87]. Foliar application of Fe chelates is an efficient method of influencing crop yield and quality, especially grain Fe contents [48,85]. Nutri-priming of seeds with Fe solution enhances Fe uptake and enrichment in crop seeds, thus improving the crop’s nutritional value and increasing its resilience to abiotic stresses [88,89].

To maximize the effectiveness of I and Fe foliar applications, it is essential to apply these nutrients during specific stages of tomato development for improved growth, yield, and fruit quality. For I, the most beneficial timing that enables effective I uptake and induces tomato fruit quality starts from developing the first fruiting branch, typically around 30–40 days after transplanting [90]. Moreover, for effective foliar application of Fe, the optimum stage is early flowering, when the plant’s nutrient demand is high [51].

To address the global consequences of I and Fe deficiency, agronomic biofortification approaches are being developed; however, to ensure their prospects of improving health, safety precautions must be kept in mind. Optimizing I bioavailability in crop plants while limiting its environmental effects involves careful management of agronomic biofortification approaches like foliar application and soil fertilization, etc. [79]. To prevent the potential health impacts linked with excessive I intake, like thyroid diseases, safe I levels must be ensured in biofortified crops [26]. According to the World Health Organization, a safe upper limit for I intake is around 1100 µg/day for adults, while the recommended dietary intake for I is approximately 150 µg/day for the general population [91]. The safe dose for I biofortification can vary depending upon the target population’s dietary intake and I deficiency level. Moreover, safety concerns are also necessary for Fe biofortification, particularly when it comes to regulating Fe levels to avoid unfavorable and toxic health complications [82]. Agronomy biofortification strategies, like soil amendments, seed priming, and Fe foliar sprays, need to be balanced. Public awareness efforts are also essential for informing the public about the advantages of consuming I- and Fe-enriched crops and addressing any concerns about their nutritional content and safety [22].

## 6. Conclusions and Future Perspectives

Our review highlights the critical public health complications caused by I and Fe deficiencies, which affect billions of people and lead to serious health problems worldwide. Agronomic biofortification arises as a useful approach to improve I and Fe contents in tomato fruit, thus addressing these deficiencies in a sustainable and accessible way. Recent developments in agronomic biofortification strategies, such as nutri-seed priming, soil supplementation, and foliar application, have shown significant potential in enhancing tomato fruit Fe and I contents. The current situation reflects limited adoption of these strategies, with research still in its early stages in some regions. To avoid potential I and Fe toxicity, particularly in youngsters and pregnant women, safety concerns must be taken seriously, which involves careful monitoring of nutrient levels in biofortified crops. To optimize I and Fe uptake and improve their bioavailability in tomato fruit, more research is required that should be focused on refining agronomic biofortification strategies, integrating agronomic methods with genetic approaches, and assessing its long-term impacts on the health of vulnerable populations. It is imperative to make the general public aware of the benefits of consuming Fe- and I-biofortified tomatoes. Consumer acceptance and uptake of Fe- and I-enriched tomatoes can be promoted through increased public awareness of their safety and health benefits. The cooperation between decision-makers, international organizations, researchers, and agricultural stakeholders determines the success of global biofortification programs. The production of Fe- and I-biofortified tomatoes will ensure that everyone has access to the nutritionally enriched food required for a healthy life.

## Figures and Tables

**Table 1:** Iodine and iron deficiency disorders and prevalence.

Micronutrient	Deficiency Disorders	Deficiency Prevalence
I	I deficiency results in goiter problems, dwarfness, cognitive impairment, hypothyroidism, infant mortality, and birth defects.	2 billion people worldwide.
Fe	Iron deficiency results in intellectual disability, birth defects, infant mortality, anemic conditions, low birth weight, and restlessness.	2 billion people worldwide. [16]

**Table 2:** Previous studies of I or/and Fe biofortification in tomato fruits.

Application Methods	Findings	Impacts on Quality Traits
Soil application of Fe	Fe enrichment up to 7.52 mg/100 g fresh weight	Improved total soluble solids and antioxidant capability [47]
Root application with KIO_3_	I enrichment up to 10 mg/kg fresh weight with minimal toxicity	Increased antioxidant ability, and secondary metabolites include flavonoids, phenolic acids, terpenoids, and alkaloids [25]
Seed priming with Fe and I	Enhanced I and Fe accumulation in tomato seeds and fruits	Enhanced antioxidant capacity and soluble solids [50]
Foliar application of I and Fe	Increased I and Fe content in tomatoes	Higher sugar content and antioxidant capacity [46]
Root application of Fe	Improved Fe content in cherry tomatoes	Increased capacity for sugars, secondary metabolites, and antioxidants [51]
Combined soil and foliar application	Effective increase in I and Fe content in tomatoes	Improved secondary metabolites, antioxidant ability, and sugars [52]
Agronomic biofortification with FeSO_4_	Significant increase in Fe content in tomatoes	Enhanced antioxidant capacity and improved total soluble solids [53]
Exogenous application of I as KIO_3_ Genetic biofortification of tomatoes to enhance Fe accumulation	Successful enrichment of I in tomato fruits Successful enrichment of Fe in tomato fruits	Improved antioxidant capacity and secondary metabolites [54] Manipulation of Fe transporters (e.g., IRT1, FRO2) and storage proteins [55]

## Data Availability

Not applicable.
